# Low origin of the radial artery: a case study including a review of literature and proposal of an embryological explanation

**DOI:** 10.1007/s12565-016-0371-9

**Published:** 2016-09-15

**Authors:** Grzegorz Wysiadecki, Michał Polguj, Robert Haładaj, Mirosław Topol

**Affiliations:** 1 Department of Normal and Clinical Anatomy, Interfaculty Chair of Anatomy and Histology, Medical University of Lodz, ul. Narutowicza 60, 90–136 Łódź, Poland; 2 Department of Angiology, Interfaculty Chair of Anatomy and Histology, Medical University of Lodz, Łódź, Poland

**Keywords:** Arterial development, Forearm, Radial artery, Variations

## Abstract

A low origin of the radial artery is a rare anatomical variation, with the incidence estimated at 0.2 %. This report presents a previously unrecorded case of an unusual distal origin of the radial artery, co-occurring with a double recurrent radial artery. The radial artery arose under the pronator teres muscle, 76 mm below the intercondylar line of the humerus. After emerging from under the tendon of the pronator teres muscle, the radial artery took a typical course and terminated in the deep palmar arch. Additionally, the double radial recurrent artery branched directly off the brachial artery, near the level of the radial neck. A well-developed muscular branch of the first radial recurrent artery ran beneath the brachioradialis muscle and supplied the brachioradialis, extensor carpi radialis longus and brevis, as well as supinator muscles. The second (accessory) radial recurrent artery took origin from the posterior aspect of the brachial artery, ran deep to the distal tendon of the biceps brachii muscle and terminated by joining the articular network of elbow. According to recent theories, the plexiform appearance of the arteries at early stages of upper limb development allows for formation of alternative pathways of blood flow, which may give rise to variations in the definitive arterial pattern.

## Introduction

Even though most anatomical variations have already been cataloged (Bergman 2011; Bergman et al. 2015), their combinations are still recorded and analyzed, as they point to complex developmental changes in anatomical relationships. Recurring reports of a given variant may imply that most human variations ‘must be in our genetic code’ (Bergman 2011; Bergman et al. 2015). Moreover, advanced diagnostic and therapeutic procedures require extensive knowledge of anatomical variations.

Variations of arteries in the arm and the cubital fossa have been well documented (Quain 1844; McCormack et al. 1953; Rodríguez-Baeza et al. 1995; Rodríguez-Niedenführ et al. 2001a, b, 2003; Nasr 2012; Klimek-Piotrowska et al. 2013; Lee et al. 2016; Kachlik et al. 2016; Piagkou et al. 2016). However, considerable deviations in the course of the radial artery (RA), located exclusively in the forearm, have been observed on only very few occasions. Rare cases of a low origin of RA combined with an atypical course of the vessel under the pronator teres muscle were reported by Quain (1844), Thomson (1884) and summarized by Bergman et al. (2015). A case of trifurcation of the brachial artery with a variant course of RA passing deep to the tendon of the pronator teres muscle was recorded by Vollala et al. (2008). There were also rare reports of hypoplasia (Gruber 1870a, b; Thomson 1884), and even the congenital absence of RA (Zheng et al. 2014). On the other hand, few studies have systematically analyzed the variant anatomy of the radial recurrent artery (RRA). Hamahata et al. (2012) and Vazquez et al. (2013) studied the arteries in the cubital fossa, with a special focus on the number, origin, course and muscle blood supply patterns of the RRA. Anatomical variations of both RA and RRA are clinically relevant, because they may influence the safety and success rate of plastic and reconstructive surgery (Bhatt et al. 2009; Hamahata et al. 2012), as well as vascular surgery and percutaneous endovascular interventions (Gaudino et al. 2014; Patel et al. 2014; Zheng et al. 2014).

This report contains an analysis of a previously unrecorded antebrachial arterial pattern. An unusual low origin of RA located under the pronator teres muscle co-occurring with the presence of a double RRA was observed and described in detail. Furthermore, significant emphasis was placed on a comparison of the similarities and differences between selected rare variations of RA in the forearm and discussing their morphogenetic background.

## Case description

During a routine dissection of an isolated right upper limb, fixed in a 10 % formalin solution, a rare variation of RA was revealed. Visual inspection of the limb allowed deformations, as well as traces of trauma or surgical procedures, to be excluded. Traditional techniques of anatomical dissection were used. Measurements of the external arterial diameter were performed with an electronic caliper (Mitutoyo Company, Kawasaki-shi, Kanagawa, Japan).

**Fig. 1a, b:**
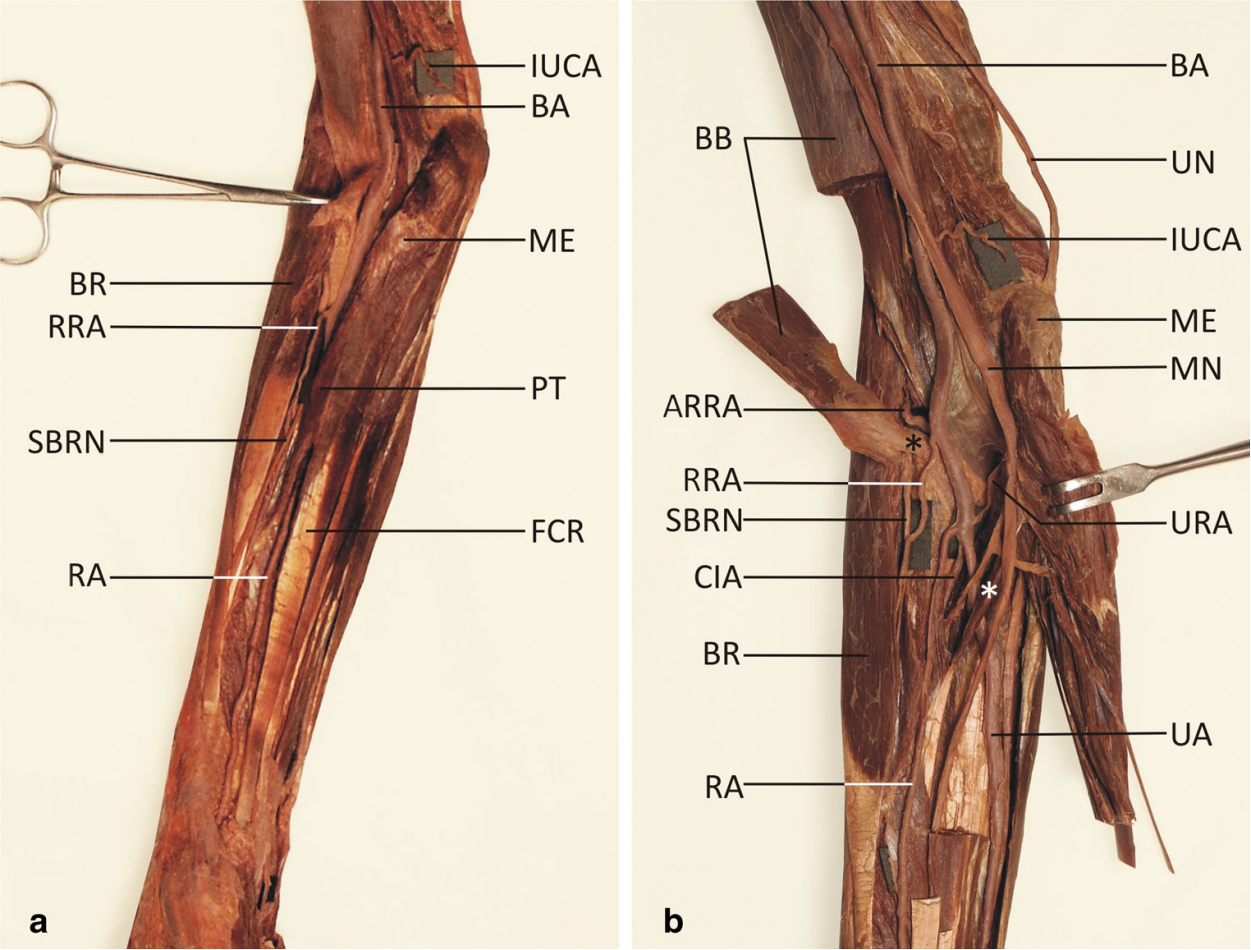
Low origin of the radial artery (RA). Stratigraphic dissection. **a** Arterial arrangement seen after removal of the skin, subcutaneous tissue and antebrachial fascia. **b** Deep dissection of the same specimen. The radial artery arises more distally than usual, under the pronator teres muscle. A well-developed muscular branch of the first radial recurrent artery (RRA) has been exposed, while a small recurrent branch of the RRA is not visible because it was accidentally damaged during specimen preparation. *ARRA* Accessory radial recurrent artery (running under the distal biceps tendon), *BA* brachial artery, *BB* biceps brachii muscle (cut and reflected), *BR* brachioradialis muscle, *CIA* common interosseous artery, *FCR* flexor carpi radialis muscle, *IUCA* inferior ulnar collateral artery, *ME* medial epicondyle, *MN* median nerve, *PT* pronator teres muscle, *RRA* muscular branch of the first radial recurrent artery, *SBRN* superficial branch of radial nerve, *UA* ulnar artery, *UN* ulnar nerve, *URA* ulnar recurrent artery, *black asterisk* distal biceps tendon (reflected), *white asterisk* accessory head of flexor pollicis longus muscle

**Fig. 2a–e:**
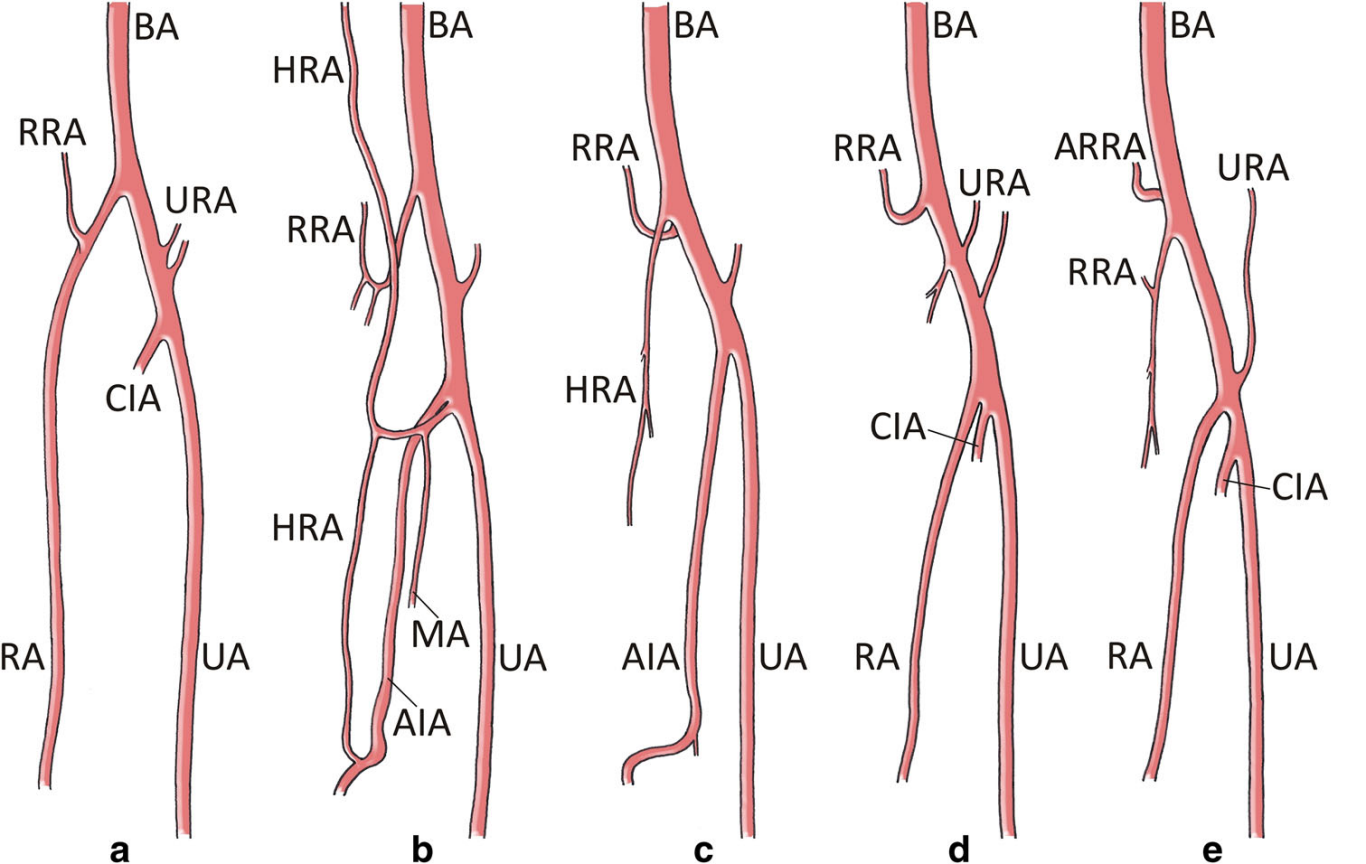
Selected anatomical variations of the RA in the forearm. **a** Typical course of the RA. **b** Rudimentary RA with three roots (anastomoting branches): the superior root from the axillary artery (not illustrated), the middle root from the median artery and the inferior root from the anterior interosseous artery. In this case the RA was present on its entire course. Described by Gruber (1870a). **c** RA replaced by an atypical branch of the anterior interosseous artery. Original Gruber (1870b) classification was maintained in the labelling of this drawing. **d** Low division and trifurcation of the brachial artery. Described by Vollala et al. (2008). **e** Low origin of the RA co-occurring with a double recurrent radial artery. Variation described in this report. *AIA* Anterior interosseous artery, *ARRA* accessory radial recurrent artery, *BA* brachial artery, *CIA* common interosseous artery, *HRA* hypoplastic (rudimentary) radial artery, *MA* persistent median artery, *RA* radial artery, *RRA* radial recurrent artery, *UA* ulnar artery, *URA* anterior and posterior ulnar recurrent arteries (**e** illustrates single ulnar recurrent artery, as described in this report)

The axillary and the brachial arteries had a normal course and branching pattern (except for the terminal part of the brachial artery). The diameter of the brachial artery, measured at the level of the intercondylar line of the humerus, was 5.14 mm. During dissection of the cubital fossa, the bicipital aponeurosis was cut and the brachial artery was exposed. The division of the brachial artery into terminal branches was not found at the typical level (Figs. 1a, b, 2e). However, a double RRA was visualized near the level of the radial neck. The first RRA (which had a diameter of 1.42 mm at the origin) arose from the brachial artery 39 mm below the intercondylar line of the humerus (Fig. 1a). It was divided into two main branches: a smaller recurrent branch, which ran anterior to the distal biceps tendon, and a well-developed muscular branch, which ran beneath the brachioradialis muscle. The muscular branch of the first RRA supplied the brachioradialis muscle, both radial wrist extensors and the supinator muscle . Additionally, at a slightly higher level, the second (accessory) RRA took origin from the posterior aspect of the brachial artery (Fig. 1b) . The accessory RRA had a diameter of 1.97 mm at the origin, ran deep to the distal tendon of the biceps brachii muscle (Fig. 1b) and terminated by joining the articular network of elbow.

At that stage of the dissection, the superficial flexor compartment was separated and reflected (Fig. 1b). During the procedure, a low origin of the RA, deep to the pronator teres muscle was found (Fig. 1b). RA arose 76 mm below the intercondylar line of the humerus, with the forearm being 27.5 cm long. The artery was well-developed and its diameter was 2.1 mm. A single ulnar recurrent artery of 1.23 mm diameter originated at approximately the same level (Figs. 1b, 2e), whereas the common interosseous artery (of 2.79 mm diameter) originated at a slightly lower level (Fig. 1b). The course and branching pattern of the common interosseous artery was typical. After emerging from under the tendon of the pronator teres muscle, RA took a normal course (Fig. 1a, b). It then gave off a small superficial palmar branch to the thenar muscles. The artery turned back at the wrist, then entered the palm by passing between the two heads of the first dorsal interosseous muscle and terminated in the deep palmar arch.

The ulnar artery ran beneath the accessory head of the flexor pollicis longus muscle (Fig. 1b), and, after pursuing a typical course, it entered the palm. The superficial palmar arch was formed exclusively by the transpalmar continuation of the ulnar artery. The diameter of RA measured at the wrist level was 2.03 mm, and the diameter of the ulnar artery at this level was 2.86 mm. In the presented case, there were no deviations from the course and branching pattern of the main nerves (median, musculocutaneous, radial and ulnar).

## Discussion

According to the classic ‘sprouting’ theory, arteries of the growing upper limb arise successively from a single trunk of the axial artery (Singer 1933; Standring 2008). At the early stages of the upper limb formation, the dominant vessel is the subclavian artery, which is continuous with the axillary, brachial and interosseous arteries developing in later stages. It is only at a further developmental stage that the ulnar and radial arteries appear (Singer 1933; Standring 2008).

Rodríguez-Baeza et al. (1995) assumed that the upper limb arteries are formed by the union of superficial and deep pathways. In this model, the superficial brachial artery is thought to be a ‘consistent embryonic vessel’ that plays an essential role during normal morphogenesis of the arteries of the upper limb. It was hypothesized (Rodríguez-Baeza et al. 1995) that, at early stages of upper limb formation, the superficial brachial artery anastomoses with a trunk for the deep origin of RA in the primitive axial artery. Therefore, the pre-anastomotic part of the superficial brachial artery usually atrophies with time (Rodríguez-Baeza et al. 1995). In a majority of cases, a typical origin of RA is established this way (Fig. 2a). Based on classic theories, as well as on the model proposed by Rodríguez-Baeza et al. (1995), the low origin of RA presented in this report may be explained by the formation of an atypical ‘root’ (trunk for origin) of RA.

**Fig. 3a, b:**
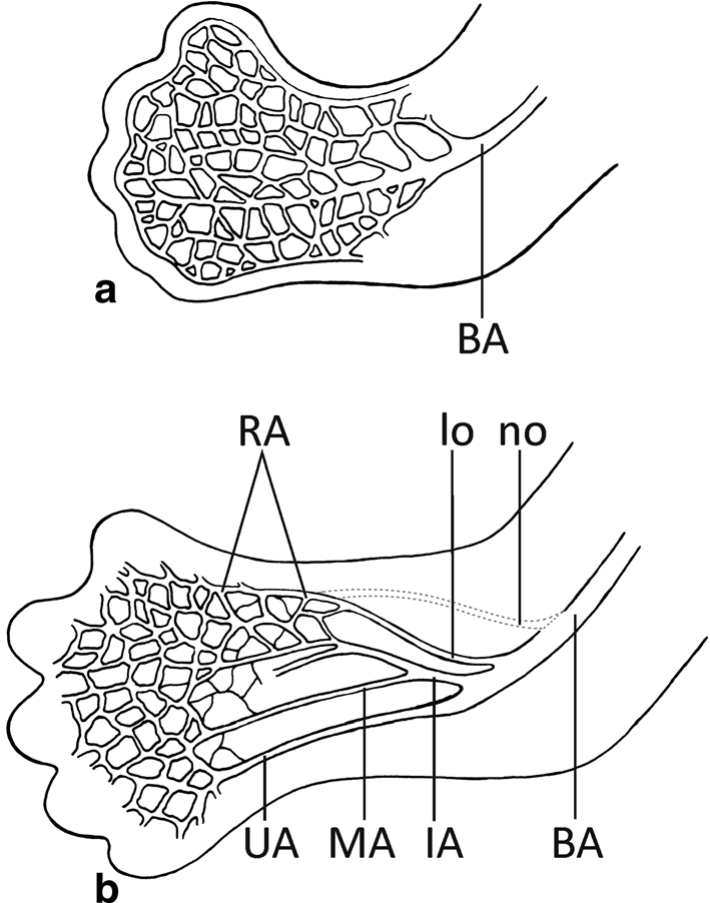
Schematic representation of the arterial remodeling in the developing upper limb between Carnegie stages 17 and 18. **a** Stage 17. At this stage, the vascular remodeling occurs at the level of the elbow, where the brachial artery branches into the capillary network. This condition may potentially allow for formation of alternative pathways of blood flow resulting in occurrence of arterial variations within the cubital fossa and the proximal part of the forearm, including a low origin of the RA. **b** Stage 18. The definitive origin of the RA is established at this stage. The distal portion of the RA remains in a capillary state, while the interosseous, median and ulnar arteries can be traced along their entire course to the hand. Drawing based on the description of Rodríguez-Niedenführ et al. (2001a, b). *BA* Brachial artery, *IA* interosseous artery, *MA* median artery, *RA* radial artery, *UA* ulnar artery, *lo* variant low origin of the radial artery, *no* potential normal origin of the radial artery (a typical course of the radial artery has been marked by *dotted lines*)

However, recent theories (Rodríguez-Niedenführ et al. 2001a, b, 2003; Vargesson 2003) suggest that the definitive arterial pattern of the upper limb is formed from the primitive capillary plexus (‘vascular labyrinth’). According to this model, the dominant vascular channels gradually differentiate as a result of capillary remodeling (Fig. 3). It is hypothesized that this mechanism of arterial development may also give rise to variations of the definitive arterial pattern (Rodríguez-Niedenführ et al. 2001a, b, 2003). As a result, some typically retained vessels may disappear or may be incompletely developed, while some collateral pathways may persist (Fig. 2b–e). Rearrangements are possible until developing vessels receive a coating of vascular smooth muscle cells (Vargesson 2003). Based on these findings, a low origin of RA may be considered a remnant of capillary anastomotic channels between the differentiating RA and the distal part of primitive axial artery (or its branches) at early stages of upper limb growth (Fig. 3). Very seldom, one of such connections might replace the normal origin of RA located in the cubital fossa (Figs. 2d, e, 3).

On the other hand, even though RA remains as a capillary plexus for a long time during morphogenesis (Fig. 3b), deviations from its typical course in the forearm are very rare (Rodríguez-Niedenführ et al. 2001a, b, 2003). Therefore, it has been suggested that many additional regulators, including genes (Bergman 2011; Aragão et al. 2016), molecular signals and growth factors (Carmeliet 2003; Vargesson 2003), interactions between endothelial and smooth muscle cells (Carmeliet 2003; Vargesson 2003), oxygenation and nutrient requirements (Carmeliet 2003), as well as hemodynamic forces (Rodríguez-Niedenführ et al. 2001a, b), could theoretically affect the definitive arterial pattern. Thus, the development of the vasculature may be defined as a series of sequential genetic and morphological events (Lin et al. 2007).

Remnants of primitive developmental relationships in forearm arteries have been documented in the literature. Some of these findings may help to understand the principles of RA formation (Fig. 2b–e). Occasional anastomotic connections between the brachioradial artery (RA of high origin) and the median or the anterior interosseous artery have been reported (McCormack et al. 1953; Rodríguez-Niedenführ et al. 2001a, b). A few anastomotic branches between RA and the superficial median artery were described by Calori (1868, cited in Bergman et al. 2015). The role of communicating channels in formation of the final configuration of arteries in the forearm is also well illustrated by a unique case (Fig. 2b) reported by Gruber (1870a), namely a rudimentary RA connected by ‘three roots’ (anastomoting branches) with the axillary artery (‘superior root’), the ‘deep’ median artery (‘middle root’) and the anterior interosseous artery (‘inferior root’). Gruber (1870b) described another case of a persistent primitive arrangement, in which, according to the original description, the distal part of a rudimentary (hypoplastic) RA was replaced by an atypical inferior branch of the anterior interosseous artery (Fig. 2c). However, according to recent knowledge (Vazquez et al. 2013), the arterial branch originally classified by Gruber (1870b) as rudimentary RA (Fig. 2c) may (if the recurrent segment were present) be considered as corresponding with RRA, which supplies the forearm muscles—similar to the case described in this report (Fig. 2e). Thus, the anterior interosseous artery may supply the hand along with a hypoplastic RA (Gruber 1870a; Thomson 1884), or may completely replace the distal part of RA (Gruber 1870b; Zheng et al. 2014).

According to Nasr’s (2012) estimates, the mean distance between the intercondylar line of the humerus and the normal origin of RA was 38.7 ± 9.5 mm in male cadavers and 36.5 ± 8.5 mm in female cadavers. A low origin of RA was reported by Quain (1844), Thomson (1884) and Vollala et al. (2008). Quain (1844) found this variation in one out of 461 limbs (~0.2 %). Quain’s (1884) finding, however, was associated with another variation, namely the existence of a *vas aberrans* proceeding from the axillary to the RA. Thomson’s (1884) report accounts for a bilateral low division of the brachial artery under the pronator teres, about ‘three inches (~76 mm) below the level of internal condyle’. However, in Thomson’s (1884) series, RA was bilaterally ‘very small’ (hypoplastic) and ‘was joined near the wrist by anterior interosseous’. By contrast, in the case reported by Vollala et al. (2008) a low origin of RA was accompanied by a trifurcation of the brachial artery into: the radial artery, the ulnar artery and the common interosseous artery (Fig. 2d), which sets that case apart from the one described in the present case study (Fig. 2e).

Since the term ‘duplication’ is defined as a condition in which an artery has two origins and one territory (Polguj et al. 2013), the reported case (Fig. 2e) was classified as ‘a double RRA’ (two arteries with different territories). Major variations of the arterial pattern in the upper limb are often associated with variable anatomy of RRAs (Vazquez et al. 2013). Moreover, if double RRAs occur, both vessels may vary in size (Vazquez et al. 2013), as in our case. According to Vazquez et al. (2013), the main RRA may originate from RA (64.8 %), the posterior radio-ulnar division (9 %), the anterior radio-ulnar division (5.4 %), the brachioradial artery (7.8 %), brachial artery (7.2 %)—as in our case, the ulnar-interosseous trunk (2.7 %) or even the interosseous trunk (0.3 %). In the latest studies conducted by Vazquez et al. (2013), the presence of a second (accessory) RRA was observed in 103 (31 %) out of 332 arms. The accessory RRA always (100 %) originated from the brachial artery, above the normal level of origin of the RA. As reported by Vazquez et al. (2013), the accessory RRA, when present, coursed behind the distal tendon of the biceps brachii muscle in all cases. The accessory RRA may supply the brachioradialis, brachialis and the biceps brachii muscles (Vazquez et al. 2013). The blood supply territory of the main RRA is larger (Vazquez et al. 2013) and comprises brachioradialis, extensor carpi radialis longus and brevis, and supinator muscles (as in the case reported in this paper). However, the muscular blood supply to extensor carpi radialis longus and brevis and supinator muscles may be provided directly from the brachial, radial, ulnar or even brachioradial arteries (Vazquez et al. 2013).

The clinical significance of a low origin of RA was described by Vollala et al. (2008). A lack of typically located initial segment of RA may affect surgical procedures using radial forearm flaps or RA grafts (Bhatt et al. 2009; Hamahata et al. 2012; Gaudino et al. 2014). Also, RA catheterization procedures may be hindered by an atypical origin and course of the vessel (Patel et al. 2014; Zheng et al. 2014).

## Conclusions

A low origin of RA is a rare anatomical variation associated with an atypical course of the vessel under the pronator teres muscle. This kind of variation may be clinically relevant, especially during reconstructions using RA, as well as during percutaneous endovascular interventions.
